# Fast detection of melamine using silver nanoparticles capped with l-cysteine functionalized carbon dots

**DOI:** 10.1039/d5ra03280f

**Published:** 2025-09-05

**Authors:** Koffi Koffi Kra Sylvestre, Dan Li, Essy Kouadio Fodjo, Aka Alla Martin, Pomi Bi Boussou Narcisse, Irié Bi Irié Williams

**Affiliations:** a Faculty of Chemical Engineering and Energy Technology, Shanghai Institute of Technology 100 Haiquan Road Shanghai 201418 China; b Laboratory of Constitution and Reaction of Matter, UFR SSMT, Felix Houphouet Boigny University Abidjan 22 BP 582 Cote d’Ivoire

## Abstract

Melamine is an additive used fraudulently to enrich foods with nitrogen, particularly in the dairy industry. It is also known as the main metabolite or degradation phytosanitary product of cyromazine. However, the numerous incidents involving living beings in aquatic environments, children and pets fed with products made from melamine in China and certain African countries have led to distrust of melamine in food. In order to ensure strong food safety and security, and good quality of the ecosystem free of melamine, it is important to design a fast, simple, reliable and efficient method for the detection of melamine. For this purpose, silver nanoparticles capped with l-cysteine functionalized carbon dots (cCDs/AgNPs) were designed for the detection of melamine. The results showed that a yellow solution of cCDs/AgNPs turns pink and gradually blue within two minutes of heating at 90 °C in the presence of melamine even at a concentration of 0.1 μg mL^−1^. This color change reflects the sensitivity of cCDs/AgNPs towards melamine. The investigation of cCDs/AgNPs-based on ultraviolet-visible spectroscopy exhibits good linearity in the range from 0.5 μg mL^−1^ to 4.5 μg mL^−1^ for melamine detection, with a detection limit of 0.03 μg mL^−1^. This method was successfully applied to determine melamine in a milk matrix, suggesting that this method can be applied for food monitoring with the aim of obtaining melamine-free food in dairy products.

## Abstract 2

Melamine is an additive used fraudulently to enrich foods with nitrogen, particularly in the dairy industry. The toxicity of melamine-enriched pet and child foods has been a source of concern these last years.

## Graphical abstract

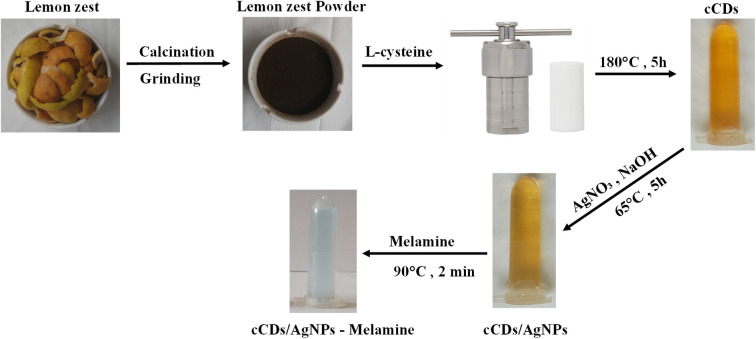

## 1. Introduction

Melamine (2,4,6-triamino-1,3,5-triazine) is a nitrogen-rich organic compound commonly used in the manufacture of fertilizers,^1^ plastics,^2^ paints^3^ and adhesives.^4^ As melamine contains a high percentage of nitrogen (66% nitrogen), its misuse in foods for protein strengthening is well known in dairy products.^5^ Between 2004 and 2008, a large number of pet owners complained about the health problems of their pets after consuming certain types of food allegedly containing melamine.^6^ Meanwhile, health scandals involving new-born and young children who allegedly consumed milk enriched with melamine have been expressed.^7^ Additionally, the World Health Organization (WHO) have confirmed the presence of melamine in a variety of dairy products following the scandal of contaminated powdered milk in some countries.^8^ Further studies reveal that feeding animals with food containing melamine led to renal failure,^9^ urolithiasis,^10^ the development of bladder stones,^11^ and kidney stones.^12^ Thus, since 2008, quantifying the presence of melamine in food products has become a global food safety concern. In order to further stem melamine contamination, some countries such as Australia, Canada, China, those of the European Union, and the USA have adopted a melamine threshold of 1 mg kg^−1^ day^−1^ for infant foods and 2.5 mg kg^−1^ day^−1^ for other dairy products and derivatives.^13^ In support of these regulations, it is essential to design fast, simple and reliable methods for melamine monitoring.

Several methods for the detection including high-performance liquid chromatography (HPLC),^14^ associated with mass spectroscopy (HPLC/MS) or ultraviolet,^15^ capillary zone electrophoresis associated with mass spectroscopy,^16^ electrochemistry or associated with potentiometric electrophoresis,^17^ and infrared^18^ have been set up. However, most of these methods suffer from complicated pretreatment as well as expensive instrument and well-trained personnel requirements. Therefore, development of facile and reliable strategies for the detection of melamine is essential. In this field, optical sensing assays with colorimetric response have sparked significant excitement due to the fact that they can offer distinct signals simply and rapidly, thus making the detection result more convincing.^19,20^ Moreover, the input species lack highly specific interactions with the nanosensor, which may also led to false-positive and false-negative results. Therefore, ensuring highly specific interactions between the introduced species, can play a key role in the rational design of a robust and reliable nanosensor.

In this work, a smart nanocomposite of l-cysteine-modified carbon quantum dots (cCDs) grafted on silver nanoparticles (cCDs/AgNPs) was fabricated in order to selectively and visually detect melamine. In its configuration, the nanocomposite gives more active sites to link with melamine nitrogen functional group or with its ring. The results show that the designed method has an excellent capacity to detect melamine. Unfortunately, some ions can affect the detection of melamine with the current designed nanosensor like other sensors. To overcome these adverse effects, an extraction of melamine in the presence of these interfering species has been introduced, and protocols based on metal complexation and/or an additional extraction procedure have been established. To the best of our knowledge, this work is the first to report this melamine detection, which proposes different extraction methods for interference-free detection. Thus, this current designed sensor can be used without any fear of interference for the detection of melamine.

## 2. Material and methods

### 2.1 Reagents

All chemicals were used without prior treatment. To carry out this work, silver nitrate (AgNO_3_, 99%) was purchased from Scharlab S.L. (Spain), sodium hydroxide (NaOH, 98%) was provided by Sigma-Aldrich (St Louis in the USA), glycine (C_2_H_5_O_2_N, 98%) and l-cysteine (C_3_H_7_NO_2_S, 98%) were acquired from Merck (Darmstadt in Germany), melamine (C_3_H_6_N_6_, 99%) was supplied by DAMAS-BETA, magnesium chloride (MgCl_2_, 99%), soluble starch ((C_6_H_10_O_5_)_*n*_, 90%) were obtained from CHEM-LAB (Belgium), aluminum sulfate (Al_2_(SO_4_)_3_, 18H_2_O, 99%), sodium sulfate anhydrous (Na_2_SO_4_, 99%), iron sulfate (FeSO_4_, *n*H_2_O, 84%) were obtained from PROLABO (France), anhydrous calcium sulfate (CaSO_4_, 98%) and anhydrous magnesium sulfate (MgSO_4_, 98%) were provided by Chem-Lab (Belgium), magnesium oxide (MgO) was synthesized according to the protocol of literature;^21^ and lemon peel samples were obtained from the fruit purchased at the local market. All experiments were done using deionized (DI) water with a resistivity of 18.25 MΩ cm.

### 2.2 Synthesis and characterization of cCDs/AgNPs

#### 2.2.1 Synthesis of l-cysteine-modified CDs

The l-cysteine-modified CDs was synthesized according to protocols described in the literature.^22^ Specifically, 5 g of lemon peels were washed and rinsed, then calcined for one hour at 200 °C in an oven. After calcination, the samples were ground and sieved to obtain brown powder. Once the powder obtained, 5 g were dispersed in 50 mL of DI water, and then 0.05 g of l-cysteine was added. The obtained mixture was transferred into a Teflon, sealed in the autoclave, and finally placed in an oven at 180 °C for five hours. After the allotted time, the autoclave was cooled to room temperature. The final solution was centrifuged, filtered to collect cCDs in colloidal form. These cCDs can have multiple surface functional groups which can be used in sensor designing.

#### 2.2.2 Synthesis of AgNPs capped by cCDs

The cCDs/AgNPs were synthesized following the procedure described by Martin and colleagues.^23^ For this synthesis, 250 μL of previous cCDs were dispersed in 200 mL of DI water. Then, under stirring, 0.01 g of AgNO_3_ was dissolved in this cCDs solution. Once the AgNO_3_ was completely dissolved, 0.025 g of NaOH was added to this mixture (which immediately appears yellow-brown in color). Then, the reaction system was heated to 65 °C under gentle magnetic stirring for 5 hours to obtain the cCDs/AgNPs in which AgNPs with their large specific surface area, were used in order to enhance the activity of cCDs functional groups.

#### 2.2.3 Characterization of cCDs and cCDs/AgNPs

The different functional groups of cCDs and cCDs/AgNPs were identified by Fourier transform infrared microscopy (FTIR) (PerkinElmer, Wellesley, USA) and by X-ray photoelectron spectroscopy (XPS) (Rigaku XSPA-400 ER, Tokyo, Japan), while the determination of UV-vis spectra was done using Optic FLAME spectrometer (Ocean Optics, Largo, USA). However, the fluorescence of cCDs and cCDs/AgNPs was assessed at 365 nm using UV lamp (Hangzhou Qiwei Instrument, Hangzhou, China), and the morphology and size distribution of cCDs/AgNPs were identified by scanning electron microscopy (SEM) (ZEISS, Oberkochen, Germany) operating at 500 kV.

### 2.3 Preparation of melamine samples

For the melamine detection using the synthesized cCDs/AgNPs, melamine stock solution was prepared by dissolving melamine in DI water. In practice, 1.2 mg of melamine was dissolved in 200 mL of DI water, and stored at 4 °C. All other melamine samples were obtained by diluting this solution.

### 2.4 Melamine detection

#### 2.4.1 Detection based on color change

In a test tube containing a sample of melamine solution (3 mL, 1.2 μg mL^−1^), 500 μL of the cCDs/AgNPs were added. This tube was then placed in a water bath to improve the reaction rate between melamine and cCDs/AgNPs. Finally, a color change was observed and UV-vis analysis was carried out to assess the effectiveness of melamine detection. To achieve higher colorimetric response of cCDs/AgNPs, the detection was performed at different temperatures to define the ideal temperature for melamine detection. Thus, three test tubes containing 3 mL of melamine sample at 1.2 μg mL^−1^ and 500 μL of cCDs/AgNPs were heated to 70 °C, 80 °C and 90 °C, respectively.

#### 2.4.2 Effect of pH

As pH can influence the functional group on the surface of the sensor and the melamine functional group, the pH of the medium was assessed. The melamine samples were prepared at different pH in the range of 3 to 12. This study allowed to define the ideal pH for optimal detection of melamine with cCDs/AgNPs.

### 2.5 Linearity of the melamine detection

To assess the linearity of the detection method, experiments were carried out to establish the calibration curve of the melamine detection method. To do this, different diluted solutions of melamine (0.5, 1.0, 1.5, 2.0, 2.5, 3.0, 3.5, 4.0, 4.5 μg mL^−1^) were used. The presence of melamine was assessed using color change of the solution at bare eye and by evaluating the variation of the UV-vis absorption of the cCDs/AgNPs in the different samples. The obtained calibration curve allows to evaluate the limit of detection and quantification limit of melamine detection using cCDs/AgNPs as a melamine sensor.

### 2.6 Interference study

To examine the specificity of the cCDs/AgNPs to detect melamine, interference of common adulterants and preservatives on detection of melamine (1.0 μg mL^−1^) were investigated. The interferents (10.0 μg mL^−1^) used for this study are some inorganic substances (Na_2_SO_4_, CaSO_4_, FeSO_4_, MgSO_4_), protein analogues (l-cystine and glycine), and carbohydrate (starch) naturally present in milk or commonly added to milk to its further enrich.

### 2.7 Extraction of melamine from powdered milk solution

The extraction of melamine from milk matrix was done using a method described by Kumar and Seth with slight modification.^24^ For this work, 2.5 g of milk matrix were dissolved in 50 mL of DI water. The solution was stirred for 5 min for homogenization. Then, 10 mL of this liquid milk solution were taken and placed in a test tube. Afterwards, 2.5 mL of magnesium oxide solution (3.6% aq.), used as anti-caking agent between melamine and proteins, carbohydrates or minerals were added to the sample and stirred vigorously for 1 min. Then, 2.5 mL of aluminum sulfate solution (7.2% aq.) was added for coagulation and flocculation of proteins and fat, and the result was stirred vigorously for another 1 min. The resulting mixture was centrifuged at 5000 rpm for 5 min. After centrifugation, the supernatant was transferred to a second test tube to adjust the pH to 8.0 with 1 M NaOH solution. This pH helps to remove the minerals present in the milk matrix in their oxide form. Once the pH was adjusted, the new mixture was filtered to collect the extract for the melamine detection. It should be noted that this extraction method is specific to milk.

## 3. Results and discussion

### 3.1 Characterization of synthesized materials

**Fig. 1:**
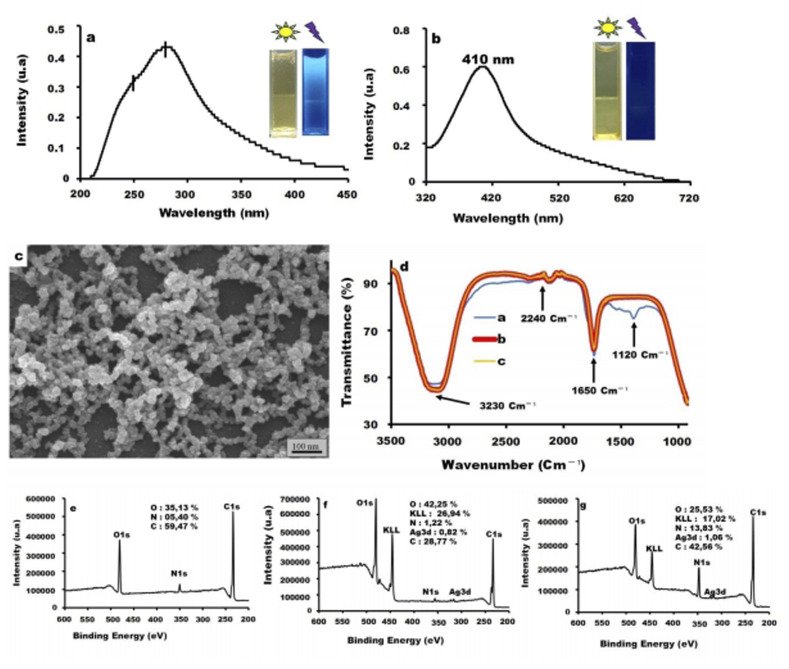
UV-vis spectrum of (a) cCDs and (b) cCDs/AgNPs. (c) SEM characterization of cCDs/AgNPs. (d) FTIR spectra of (D1) cCDs, (D2) cCDs/AgNPs and (D3) cCDs/AgNPs-melamine. XPS spectrum of (e) cCDs, (f) cCDs/AgNPs, and (g) cCDs/AgNPs-melamine.

In this work, the synthesized nanoparticles were characterized by different techniques in order to confirm the functionalized CDs and CDs/AgNPs by the hydrothermal method from lemon peel powder (Fig. 1). As shown in Fig. 1a, the UV-vis absorption spectrum of cCDs presents two characteristic peaks appearing at 250 nm and 280 nm, which are typical for CDs. Indeed, the absorption peak observed at 280 nm corresponds to the π–π* transitions in the C=C bonds of sp^2^ hybridization, while the shoulder appearing at 250 nm might be associated with the n–π* transitions of the C–O or C–N bonds, suggesting that the synthesized cCDs possessed carbonyl, amine, amide, and alcohol groups. Similar results were obtained in the literature.^25^ Furthermore, the cCDs emit a blue fluorescence under UV irradiation at 365 nm, indicating the presence of chromophore and conforming the existence of above functional groups. Moreover, the obtained cCDs were used as stabilizing and functionalizing agents for the synthesis of silver nanoparticles (cCDs/AgNPs). The analysis of synthesized cCDs/AgNPs containing in the colloidal solution (pH 9), was done using a UV-vis spectrophotometer and SEM. The UV-vis analysis indicated a maximum absorption peak around 410 nm (Fig. 1b) which is a characteristic peak of silver nanoparticles (AgNPs), giving thus evidence of the formation of AgNPs. Furthermore, the analysis of cCDs/AgNPs by SEM (Fig. 1c) reveals the presence of stacked and polydispersed spherical nanoparticles (Fig. S1). However, UV irradiation (365 nm) of the colloidal solution of cCDs/AgNPs shows the loss of the fluorescence of these cCDs/AgNPs. This loss of fluorescence of cCDs could be due to the breakup of the chromophore function induced by conjugation of C=C double bonds following the intrusion of AgNPs into the structure of cCDs or the oxidation of some of the functional groups on the peripheral cCDs structure during Ag^+^ reduction, thereby deleting fluorescence. Furthermore, according to the literature, high pH can dissociate CDs by diluting the amide and carboxylic bonds, leading thus to CDs deactivation.^26^

**Scheme 1:**
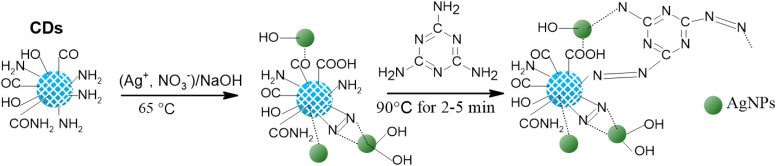
Mechanism of designing of cCDs/AgNPs as sensor and the process of melamine detection.

In order to further analyze chemical and structural information, FTIR characterization of cCDs, cCDs/AgNPs, and cCDs/AgNPs-melamine were carried out (Fig. 1d). As exhibited, except the spectrum of cCDs which shows a difference in the spectral range between 1200 cm^−1^ and 1500 cm^−1^, cCDs/AgNPs, and cCDs/AgNPs-melamine display the same profile, indicating that all synthesized nanostructures (cCDs, cCDs/AgNPs, and cCDs/AgNPs-melamine) have almost common functional groups on their surface. Indeed, the spectra display a broad band between 3380 cm^−1^ and 3230 cm^−1^ which results from the stretching vibrations of –OH, –NH, and C–H from sp^2^ and sp, while a strong vibration peak at 1640 cm^−1^ is mainly associated with the stretching vibrations of C=C, C=N, and C=O. As for the peaks observed at 2240 cm^−1^ and around 1880 cm^−1^, they are associated with C≡N or C≡C, and O=C–OH stretching vibrations. Furthermore, the additional band between 1200 cm^−1^ and 1500 cm^−1^ in cCDs spectrum (Fig. 1d; D1) can be attributed to both bending vibrations of C–H, stretching vibrations of C–O, C–N and C–C, and ring vibrations, confirming the planar structure and surface passivation of cCDs. All these results indicate that the surface of these structures are rich in alkenes, amine, amide, imine, alcohol, carbonyl and amide functional groups.^27^ Furthermore, the disappearance of the band between 1200 cm^−1^ and 1500 cm^−1^ in IR spectra of cCDs/AgNPs or cCDs/AgNPs-melamine compared to the spectrum of cCDs suggests that AgNPs have linked to cCDs through N, O, and the rings of cCDs. However, the lower absorption peak of cCDs compared with those of cCDs/AgNPs and cCDs/AgNPs-melamine in the range between 3380 cm^−1^ and 3230 cm^−1^ illustrates that the available amount of O–H of carboxylic and –NH of amide have increased. This effect may be explained by the oxidation of certain functional groups on the cCDs such as alcohol to carbonyl and amine to imine or azo groups following the reduction of Ag^+^ ions, and especially the release of new functional groups under the presence of hydroxide ions from alkaline solution which can react with defective rings and passivate AgNPs.^28^ These result may also suggest that AgNPs linked to cCDs through oxygen or nitrogen atoms. However, the similarity in the TFIR profile of cCDs/AgNPs and cCDs/AgNPs-melamine rich in amine, might be explained by the bonds between cCDs/AgNPs and melamine. The fact that there is no modification in the absorption peaks suggests that the amine functions from melamine and cCDs/AgNPs were used for the bonds between melamine and cCDs/AgNPs,^29^ as illustrated in Scheme 1.

To further confirm the surface functional groups, the XPS characterization of cCDs, cCDs/AgNPs, and cCDs/AgNPs-melamine were carried out (Fig. 1e–g and S2–S5). As illustrated in Fig. 1e–g, three main peaks associated with O1s around 530 eV, N1s around 400 eV, and C1s around 284 eV are clearly distinguished for the three structures (cCDs, cCDs/AgNPs and cCDs/AgNPs-melamine). Besides these peaks, two other small peaks around 370 eV associated to Ag3d, close to each other are observed for cCDs/AgNPs, and cCDs/AgNPs. These results suggest that functional groups containing carbon, oxygen, and nitrogen are present on the cCDs surface while functional groups containing carbon, oxygen, silver and nitrogen are present on the surface of cCDs/AgNPs and cCDs/AgNPs-melamine. From the survey for each atom (Fig. S2–S5), it is obvious by the different intensities that the surface of cCDs is more rich in carbon atom than other surfaces, in which there is as much carbon in cCDs/AgNPs as in cCDs/AgNPs-melamine. Looking closely, cCDs surface contains nitrogen in pyridinic-like or graphic-like environment (N in aromatic ring) confirming the planar structure of cCDs as suggested in IR results. Furthermore, cCDs/AgNPs surface is the richest in nitrogen atom indicating that the presence of AgNPs have enhanced nitrogen presence on the surface of cCDs/AgNPs and lost its oxygen richness as expected with cCDs. This effect suggests that AgNPs are mostly linked to cCDs through oxygen. Moreover, in the case of cCDs/AgNPs-melamine, the surface displays more oxygen atom than the other structures (cCDs and cCDs/AgNPs). This indicates that more nitrogen functional groups have been removed from cCDs/AgNPs-melamine surface due to the formation of N=N bonds with amine from melamine and another amine from cCDs/AgNPs. In more detail, as illustrated in Fig. S2, cCDs, cCDs/AgNPs, and cCDs/AgNPs-melamine spectra exhibit the same trend with a slight shift to higher binding energy of the peaks. This result indicates that these structures have the similar carbon functional groups such as carbonyl, amide, amine, alkene, hydroxide, and ether with different proportions according to the peak intensity. However, in Fig. S3, cCDs show one band from 528.9 eV to 535 eV which is characteristic functional group involving oxygen (carbonyl, oxime, alcohol, …). Besides this peak, another band between 534.2 eV and 537.7 eV is observed in the case of cCDs/AgNPs and cCDs/AgNPs-melamine, this band confirms the above functional groups and the presence of Ag–O on the surface of cCDs/AgNPs and cCDs/AgNPs-melamine. Meanwhile, in Fig. S4, one can see the difference in the spectral profile of the different structures. Like in the O1s profile, cCDs show only one band from 398 eV to 403 eV attributed to C–N, N–O, N–H, N–N and N in rings binding energy. cCDs/AgNPs display three band energy, one from 398 eV to 401 eV ascribed as C–N, N–O, N–H, N–N functional groups, the second which is a peak energy around 403 eV attributed to N–O and the last one from 405.5 to 408 which is associated to NO_3_^−^ (the ions from silver nitrate) and to characteristic binding energy peak of melamine. Moreover, in Fig. S5, two small peaks close to each other around 373 eV and 367 eV attributed to Ag3d can be observed. These two peaks are 6 eV apart from each other, suggesting that these peaks are due to the spin-orbital splitting of Ag3d which correspond to Ag3d_3/2_ and Ag3d_5/2_ core levels, respectively, as suggested the literature.^30^ All these results are in good agreement with those obtained with TFIR.

### 3.2 Detection of melamine using cCDs/AgNPs as sensor

#### 3.2.1 Sensitivity of cCDs/AgNPs

**Fig. 2:**
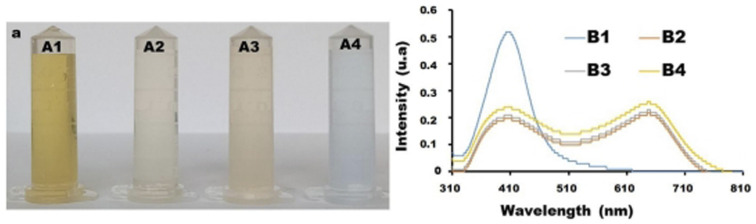
(a) Effect of temperature at (A1) 28 °C, (A2) 70 °C, (A3) 80 °C and (A4) 90 °C on the detection of 3 mL of 1.2 μg per mL melamine with 300 μL of cCDs/AgNPs. (b) Associated UV-vis spectrum for the detection of melamine at 28 °C (B1), 70 °C (B2), 80 °C (B3) and 90 °C (B4).

To study the effect of temperature on the sensitivity of melamine *versus* the synthesized cCDs/AgNPs, different temperatures were evaluated (Fig. 2). In the presence of melamine the cCDs/AgNPs-melamine mixture turns from yellow to pink at 70 °C and 80 °C (Fig. 2a, A2, and A3) and to blue at 90 °C (Fig. 2a, A4) within 2 min using water bath. This color change is characterized by a new peak at 650 nm as shown in Fig. 2b, demonstrating that the interaction of cCDs/AgNPs and melamine is temperature-dependent. Indeed, at temperature around 28 °C, the color of the solution does not change throughout the duration of the experiments, which indicates that the reaction undergoes slowly and would take long time to be realized (Fig. 2b, B1), but when the temperature increases till 70 °C, the color change is observable. It should be noted that the pink color obtained between 70 °C and 80 °C is an intermediate color which finally changes to blue over a time dependent on the experimental temperature. This result illustrates that the interaction between cCDs/AgNPs and melamine is temperature-dependent and high temperature improves the kinetics of the reaction.^23^

#### 3.2.2 Effect of pH on the melamine detection

**Fig. 3:**
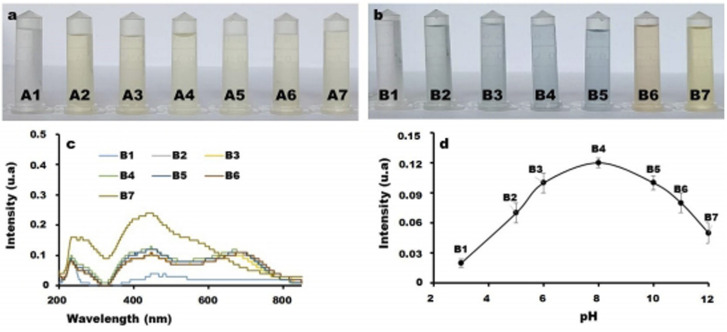
(a) and (b) Effect of pH on cCDs/AgNPs in the absence and presence of melamine (3 mL, 1.2 μg mL^−1^) for 2 min of heating at 90 °C and at different pH: (A1 and B1) pH 3, (A2 and B2) pH 5, (A3 and B3) pH 6, (A4 and B4) pH 8, (A5 and B5) pH 10, (A6 and B6) pH 11 et (A7 and B7) pH 12. (c) UV-vis spectra cCDs/AgNPs-melamine mixture at different pH. (d) pH detection trend curve obtained from (e) at wavelength *λ* = 410 nm.

As the functional groups on cCDs/AgNPs and on melamine are dependent on the pH, the impact of pH on melamine detection using cCDs/AgNPs as sensor was studied over the pH range from 3 to pH 12 and at wavelength *λ* = 410 nm. As exhibited in Fig. 3, when the pH = 3 (Fig. 3a and b), no color change of cCDs/AgNPs and melamine mixture was observed. This behavior might be due to the high concentration of H^+^ ions which may reacted with cCDs, destroying conjugated C=C bonds in CDs or interacting with the heteroatom on cCDs borders. Meanwhile, when the pH is greater than 3 but lower than 11, cCDs/AgNPs solution turned from yellow to pink and finally to blue in the presence of melamine, observable at bare eye. In this pH range, the presence of the different solution ions (H^+^ and OH^−^) has moderated influence on the detection of melamine by cCDs/AgNPs, suggesting that this range is convenient for the detection of melamine. However, according to Fig. 3d, the optimum pH is 8, beyond, the sensor gradually loses its sensitivity with melamine. This behavior of the sensor at higher pH than 8 is related to the fact that the OH^−^ ions have sufficient concentration to compete with melamine on the surface of the sensor. Thus, the pH of remain experiments for the detection of melamine using the cCDs/AgNPs sensor is set at pH 8.

#### 3.2.3 Calibration and limit of detection

**Fig. 4:**
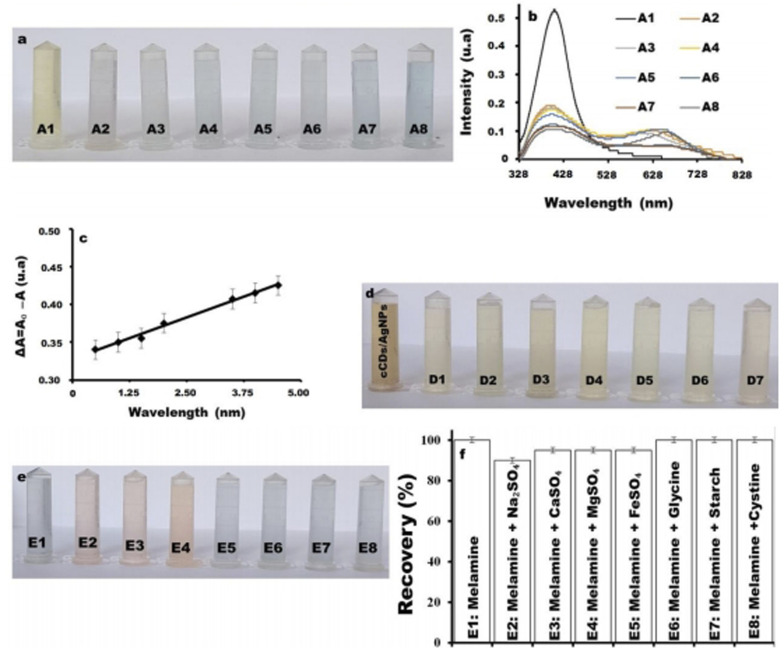
(a) Detection of melamine for 2 min of heating at 90 °C in different concentrations (A1) 0 μg mL^−1^, (A2) 0.5 μg mL^−1^, (A3) 1.0 μg mL^−1^, (A4) 1.5 μg mL^−1^, (A5) 2.0 μg mL^−1^, (A6) 3.5 μg mL^−1^, (A7) 4.0 μg mL^−1^ and (A8) 4.5 μg mL^−1^ at pH8. (b) UV-vis absorption spectrum of cCDs/AgNPs in each sample from (a). (c) Calibration curve of melamine detection collected from (b) using the difference of absorbance (without and with melamine) *versus* the concentration. (d) Interference tests on cCDs/AgNPs with 1.0 μg mL^−1^ of (D1) Na_2_SO_4_, (D2) CaSO_4_, (D3) MgSO4, (D4) FeSO_4_, (D5) glycine, (D6) starch and (D7) cystine. (e) Melamine (1.0 μg mL^−1^) detection in the presence of 10.0 μg mL^−1^ interferents (E*i* is the correspondent of D*i* with the presence of melamine). (f) Melamine recovery diagram obtained from (e).

To establish the calibration range for the determination of unknown amount of melamine in samples, different concentrations of melamine solutions ranging from 0.5 μg mL^−1^ to 4.5 μg mL^−1^ were prepared, and 300 μL of cCDs/AgNPs were added to each solution (Fig. 4a). As shown in Fig. 4b, the increase in melamine concentration leads to the decrease in the UV-vis absorption peak of cCDs/AgNPs. The absorbance Δ*A* = *A*_0_ − *A* (*A*_0_, the absorbance without melamine, and A, the absorbance in the presence of melamine at the concentration C) of cCDs/AgNPs at different concentrations of melamine and at wavelength *λ* = 410 nm indicates a linear trend (Fig. 4c) with the equation:Δ*A* = 0.0219 × *C*(μg mL^−1^) + 0.3278 with *R*^2^ = 0.9925.

Using the slope (*σ*) and the standard deviation (*Φ*) of the calibration curve, the calculated limit of detection, and the limit of quantification are 0.03 μg mL^−1^ and 0.10 μg mL^−1^, respectively.

#### 3.2.4 Selectivity of the detection method

As the milk matrix can contain multiple substances to improve either its food properties or its conservation properties, the selectivity of cCDs/AgNPs sensor was studied. This study is focused on the reliability tests to reduce undesired effects of certain substances likely to be present with melamine in a real milk matrix. For these tests, inorganic compounds, namely sodium sulfate (Na_2_SO_4_), calcium sulfate (CaSO_4_), magnesium sulfate (MgSO_4_), iron sulfate (FeSO_4_), and carbohydrate, and protein analogues (starch, cystine and glycine) were used. In the conditions of precipitation of metallic minerals (pH > 8) and detections, cCDs/AgNPs are almost insensitive of the different interferents used in this study with a slight sensitivity with cystine (Fig. 4d) and with recovery rate between 95% and 105% (Fig. 4e and f). However, in the solutions of CaSO_4_, MgSO_4_ and FeSO_4_, the detection of melamine is expressed by a pink coloration unlike the other solutions which display a blue coloration. This difference in coloration can be explained by the persistence of the residues from Ca^2+^, Mg^2+^ and Fe^2+^ minerals, which could induce a complexation of cCDs/AgNPs favored by the presence of some functional groups such as carbonyl, amine and amide groups on the surface of the sensor. The above results indicate that the cCDs/AgNPs nanosensor can be effectively used for the monitoring of melamine in dairy products without fear of interference.

### 3.3 Detection of melamine in a real environment

**Fig. 5:**
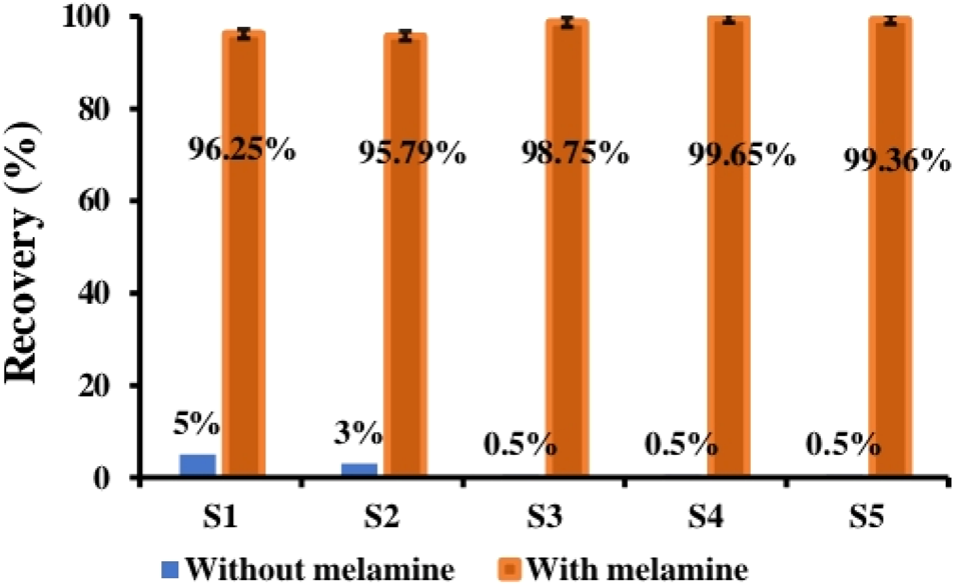
Melamine recovery diagram in different milk powder samples: E1: extract from” Bonnet rouge”, E2: extract from “Nido”, E3: extract from “full cream milk powder Lp”, E4: extract from Laity sample and E5: extract from “Top-Lait”.

The application of cCDs/AgNPs in real matrices was carried out using five different samples (Bonnet Rouge, Nido, Lait en Poudre (Lp), Laity, and Top-Lait) of milk powder widely sold on the national market. For the purpose of this study, these samples were contaminated with melamine (5 mg melamine added to 2.5 g milk powdered, either 2 mg g^−1^), and the extraction were done as ascribed in the Experiment section. The detection processes were performed using cCDs/AgNPs nanosensor. The results of this study are presented in Fig. 5.

In this study, the melamine recovery rate varies from 95% to 99%. Furthermore, the evaluation of the relative standard deviation (RSD) resulting of application of cCDs/AgNPs in a real matrix is summarize in Table 1.

**Table 1:** Recovery of melamine in a real environment

Added (μg mL^−1^)	Found (μg mL^−1^)	RSD (%)
0.50	0.49 ± 0.01	2.0
1.00	0.98 ± 0.07	7.1
3.00	2.92 ± 0.25	8.6
4.00	3.86 ± 0.14	3.6

This study shows that cCDs/AgNPs nanosensor can be applied in real matrix with RSD lower than 10.0%. Thus, cCDs/AgNPs nanosensor is an excellent detection tool for melamine with good precision.

### 3.4 Comparison with other detection methods

The usability of the detection method proposed by our work has been assessed by a comparative study with other published results concerning the detection of melamine (Table 2).

**Table 2:** Comparison with other published methods for the detection of melamine^a^

Analytical method	LOD	Linear range	Reference
Colorimetric (AuNPs)	0.05 μg mL^−1^	0.10–2.00 μg mL^−1^	24
EIA	0.02 μg mL^−1^	0.02–0.50 μg mL^−1^	31
GC-MS	0.10 μg mL^−1^	0.10–1.00 μg mL^−1^	32
SERS-AuNPs	0.10 μg mL^−1^	0.10–0.20 μg mL^−1^	33
Immunoassay	0.50 μg mL^−1^	0.50–1.00 μg mL^−1^	34
HPLC	0.10 μg mL^−1^	1.00–80.0 μg mL^−1^	35
HPLC/UV/MS	0.02 μg mL^−1^	0.04–1.00 μg mL^−1^	36
Reverse-phase HPLC	0.10 μg mL^−1^	5.00–40.0 μg mL^−1^	37
HILIC-UV	0.02 μg mL^−1^	0.00–0.50 μg mL^−1^	37
Colorimetric (AgNPs)	0.08 μg mL^−1^	0.08–0.25 μg mL^−1^	38
Colorimetric (cCDs/AgNPs)	0.03 μg mL^−1^	0.50–4.50 μg mL^−1^	This work

a GC-MS, gas chromatography coupled with mass spectrometry; EIA, enzyme-linked immunosorbent assay; SERS, surface enhanced Raman spectroscopy; HPLC, high performance liquid chromatography; HILIC-UV, hydrophilic interaction liquid chromatography with UV.

This comparative study shows that the limit of detection (LOD) of this new approach is in the same trend as those obtained with GC-MS or colorimetry using AuNPs as sensors. In addition of the detection at bare eye, the interference which constitutes one of the main drawbacks of these detection techniques is overcome with the current nanosensor. Thus, the proposed method is a highly competitive method for the monitoring of melamine levels in food products. Moreover, its simplicity, sensitivity, precision, low cost, and excellent recovery rate make it an interesting alternative for the analysis of food products.

## 4. Conclusion

This method uses a colorimetric sensor consisting of a silver nanoparticle capped with carbon dots functionalized by l-cysteine: cCDs/AgNPs. This sensor was applied to the discrimination and detection of melamine. It follows that in the presence of melamine, the solution of cCDs/AgNPs sensor heated at 90 °C, change from yellow to blue with a response time of 2 min. This change of color can be observed at bare eye without any means of analysis tool. The results indicate that a LOD of approximately 0.03 μg mL^−1^ can be obtained. This result is in the same order, or even lower than those obtained with certain traditional analytical methods, suggesting the sensitivity of the designed nanosensor. Furthermore, the reliability and performance of this method especially with its obvious sensitivity, its recovery rate greater than 95%, and its acceptable LOQ, make cCDs/AgNPs sensor a promising alternative in the quest for food safety through the monitoring of melamine levels in dairy products. These striking properties substantially make this cCDs/AgNPs-based colorimetric assay more convenient and more readily adopted for on-site fast monitoring of dairy products and melamine-related toxicology.

## Author contributions

Authors contributed equally.

## Conflicts of interest

There are no conflicts to declare.

## Supplementary Material

RA-015-D5RA03280F-s001

## Data Availability

No primary research results, software or code have been included and no new data were generated or analysed as part of this review. Supplementary information is available. See DOI: https://doi.org/10.1039/d5ra03280f.
