# Supplementary material for: Fast detection of melamine using silver nanoparticles capped with l-cysteine functionalized carbon dots
Source: RSC Adv. 2025 Sep 5;15(39):32031–40. doi: 10.1039/d5ra03280f (PMC12412118; doi:10.1039/d5ra03280f)
Supplement: RA-015-D5RA03280F-s001 [file RA-015-D5RA03280F-s001.pdf]

Please do not adjust margins

SUPPORTING INFORMATION

Fast Detection of Melamine using Silver Nanoparticles Capped with L-Cysteine Functionalized Carbon dots

Received 00th January 20xx,
Accepted 00th January 20xx

DOI: 10.1039/x0xx00000x

Koffi Koffi Kra Sylvestre[a,b], Dan Li[a], ESSY Kouadio Fodjo*[b], Aka Alla Martin[b], Pomi Bi Boussou Narcisse[b], Irié Bi Irié Williams[b]

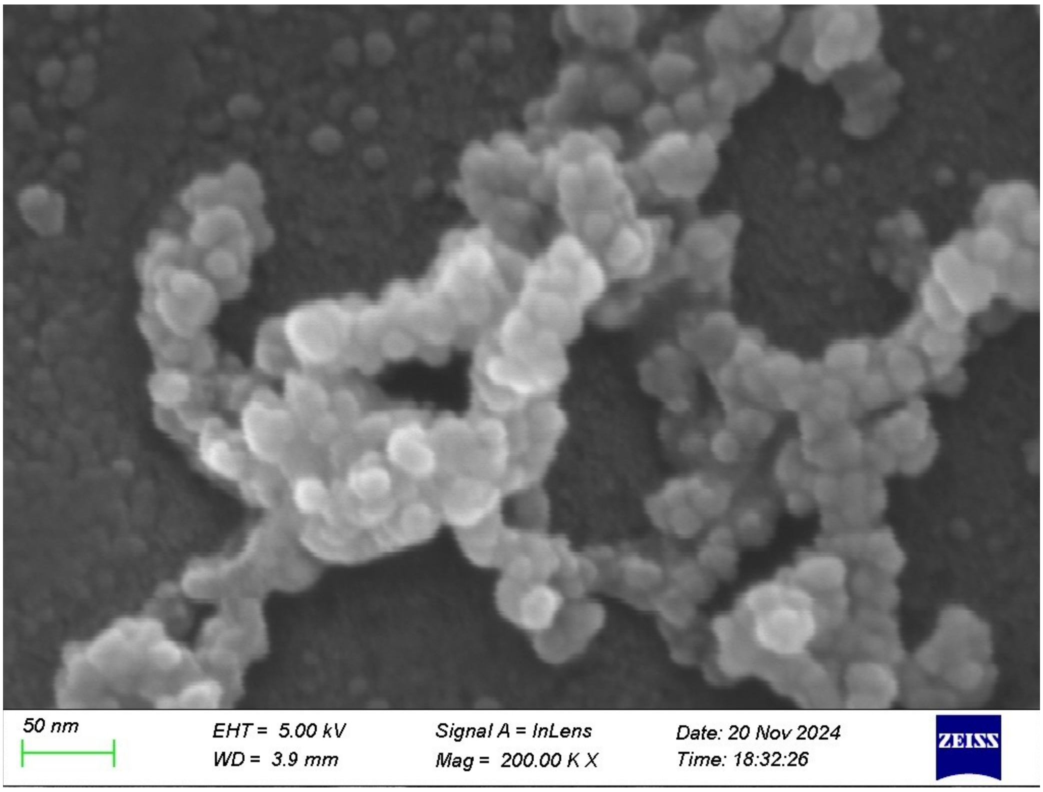

Fig. S1 SEM characterization of cCDs/AgNPs

a. *School of Chemical and Environmental Engineering, Shanghai Institute of Technology, 100 Haiquan Road, Shanghai 201418, PR China.*
b. *Laboratory of Constitution and Reaction of Matter, UFR SSMT, Felix Houphouet Boigny University, 22 BP 582 Abidjan 22, Cote d'Ivoire.*
† *Corresponding author:*
ESSY Kouadio Fodjo
ORCI: 0000-0002-7915-7216.
Supplementary Information available: [details of any supplementary information available should be included here]. See DOI: 10.1039/x0xx00000x

Please do not adjust margins

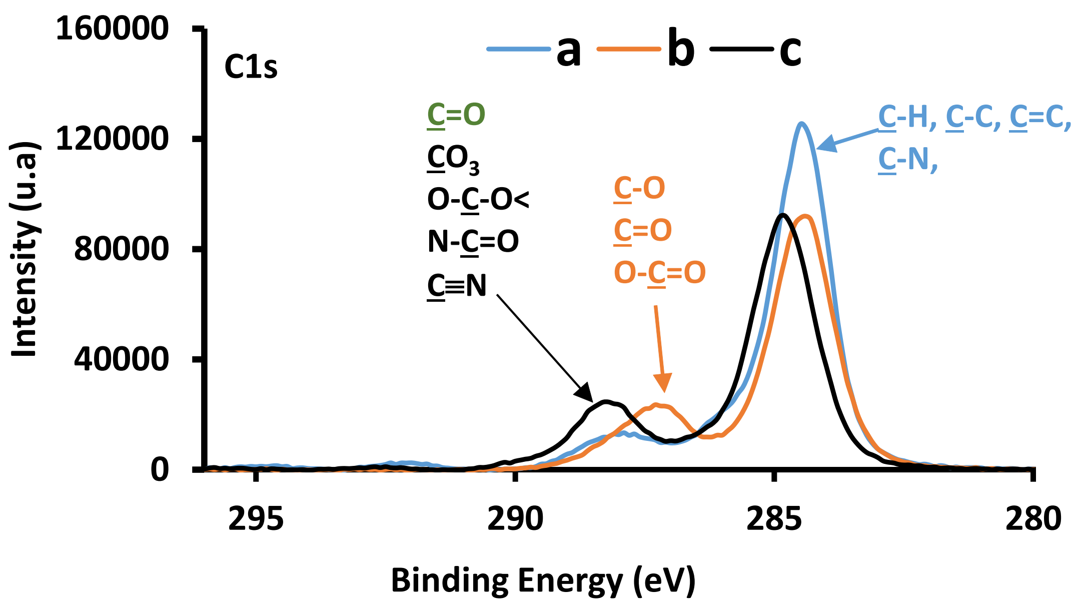

Fig. S2 XPS spectra of C1s for (a) cCDs, (b) cCDs/AgNPs and (c) cCDs/AgNPs-melamine

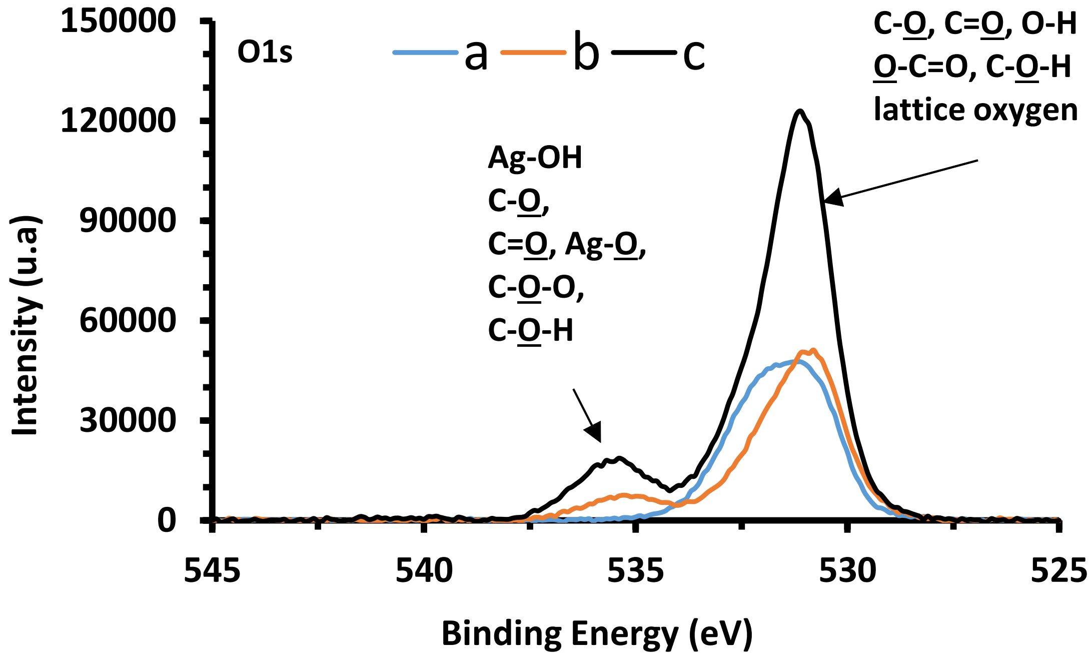

Fig.S3 XPS spectra of O1s for (a) cCDs, (b) cCDs/AgNPs, and (c) cCDs/AgNPs-melamine

Please do not adjust margins

Please do not adjust margins

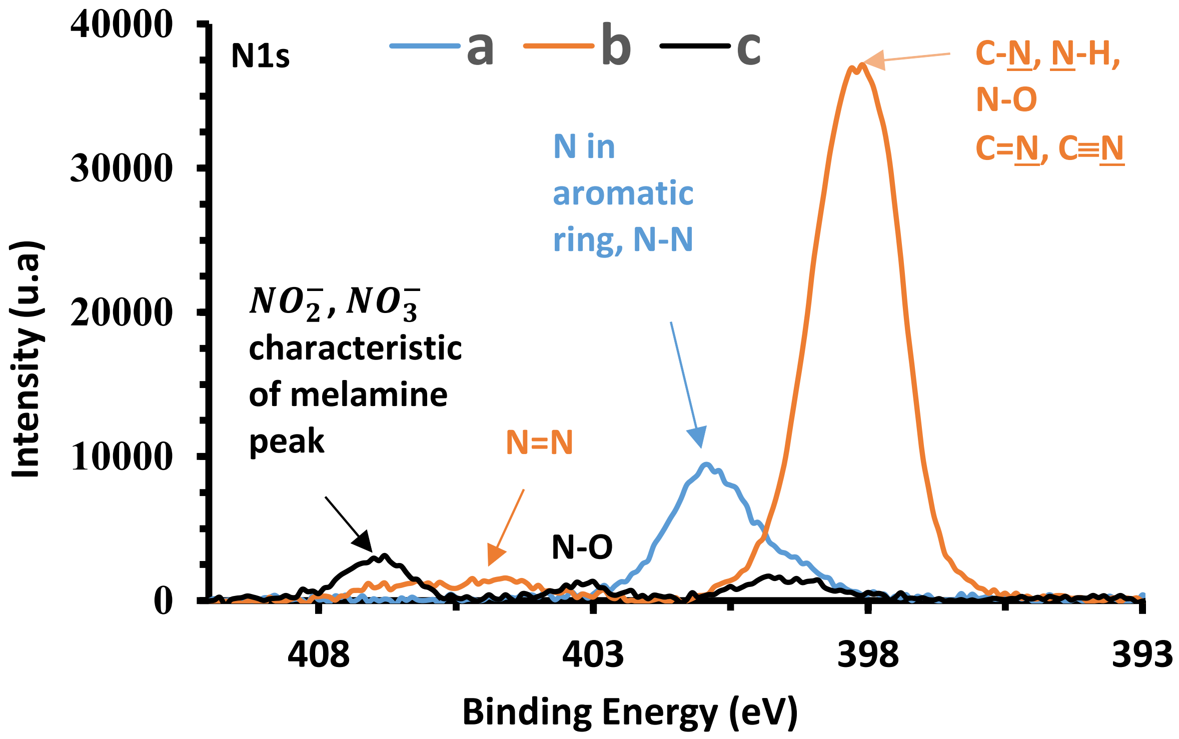

Fig. S4 spectra of N1s for (a) cCDs, (b) cCDs/AgNPs, and (c) cCDs/AgNPs-melamine

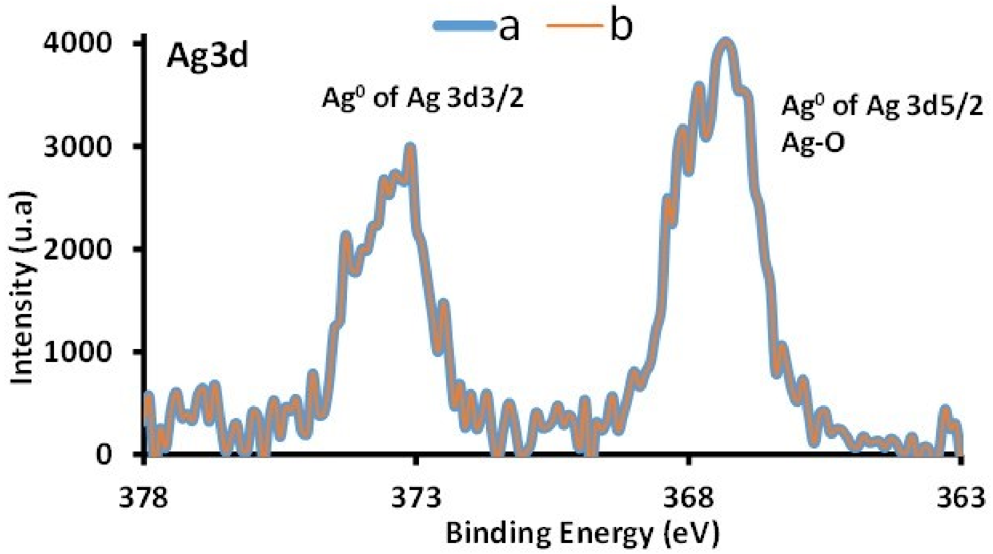

Fig. S5 XPS spectra for cCDs/AgNPs and cCDs/AgNPs-melamine

Please do not adjust margins
